# Latest Knowledge on the Role of Vitamin D in Hypertension

**DOI:** 10.3390/ijms24054679

**Published:** 2023-02-28

**Authors:** Niklas S. Jensen, Markus Wehland, Petra M. Wise, Daniela Grimm

**Affiliations:** 1 Department of Biomedicine, Aarhus University, Ole Worms Allé 4, 8000 Aarhus, Denmark; 2 Department of Microgravity and Translational Regenerative Medicine, University Clinic for Plastic, Aesthetic and Hand Surgery, Otto von Guericke University, Universitätsplatz 2, 39106 Magdeburg, Germany; 3 Research Group “Magdeburger Arbeitsgemeinschaft für Forschung unter Raumfahrt- und Schwerelosigkeitsbedingungen” (MARS), Otto von Guericke University, 39106 Magdeburg, Germany; 4 The Saban Research Institute, Children’s Hospital Los Angeles, University of Southern California, 4650 Sunset Blvd, Los Angeles, CA 90027, USA

**Keywords:** vitamin D, vitamin D deficiency, hypertension, antihypertensive treatment, supplement, clinical trials, molecular mechanisms

## Abstract

Hypertension is the third leading cause of the global disease burden, and while populations live longer, adopt more sedentary lifestyles, and become less economically concerned, the prevalence of hypertension is expected to increase. Pathologically elevated blood pressure (BP) is the strongest risk factor for cardiovascular disease (CVD) and related disability, thus making it imperative to treat this disease. Effective standard pharmacological treatments, i.e., diuretics, angiotensin converting enzyme (ACE) inhibitors, angiotensin receptor blocker (ARBs), beta-adrenergic receptor blockers (BARBs), and calcium channel blockers (CCBs), are available. Vitamin D (vitD) is known best for its role in bone and mineral homeostasis. Studies with vitamin D receptor (VDR) knockout mice show an increased renin–angiotensin–aldosterone system (RAAS) activity and increased hypertension, suggesting a key role for vitD as a potential antihypertensive agent. Similar studies in humans displayed ambiguous and mixed results. No direct antihypertensive effect was shown, nor a significant impact on the human RAAS. Interestingly, human studies supplementing vitD with other antihypertensive agents reported more promising results. VitD is considered a safe supplement, proposing its great potential as antihypertensive supplement. The aim of this review is to examine the current knowledge about vitD and its role in the treatment of hypertension.

## 1. Introduction

High blood pressure (hypertension) is a serious risk factor for cardiovascular diseases, such as coronary artery disease, myocardial infarction, or stroke, if untreated [1]. Study results revealed that vitamin D deficiency ameliorates the development of hypertension (HT) [1,2]. Vitamin D deficiency (25-OH-D < 30 ng/mL) is an independent risk factor for high blood pressure and is involved in the promotion of cardiovascular mortality [3].

**Figure 1:**
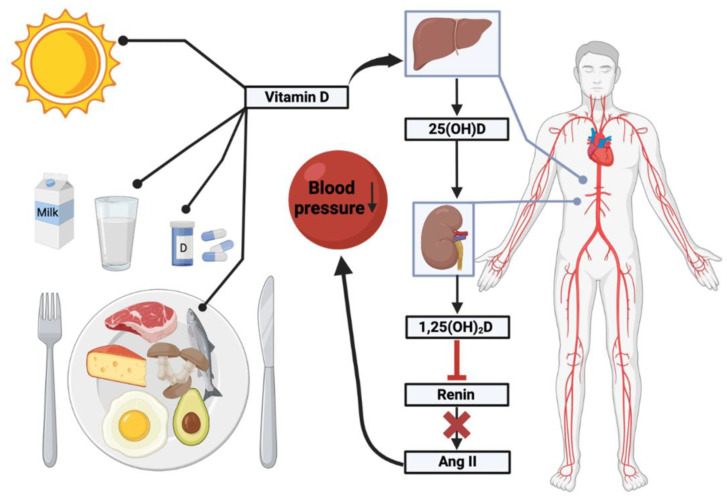
Illustrated abstract of Vitamin D and its role in hypertension. This figure was made using Biorender.com, agreement number MD24V0MZH0.

In autumn and winter, until the beginning of April, the sunlight intensity in Europe is not sufficient for our body to synthesize sufficient vitamin D, which results in vitamin D deficiency (Figure 1). It is well known that vitamin D is involved in calcium homeostasis and bone metabolism, and that supplementation in the elderly can reducing the fracture risk [4].

Epidemiological and clinical studies demonstrated an association between inadequate exposure to sunlight, vitamin D deficiency, and hypertension or increased plasma renin activity [1,2,3]. On average, blood pressure values are lower in the summer than in winter [5,6,7].

This concise review summarizes the latest knowledge about vitamin D deficiency and arterial hypertension. The available literature published from 2017–2022 is evaluated and reviewed in the following chapters.

## 2. Methods

**Figure 2:**
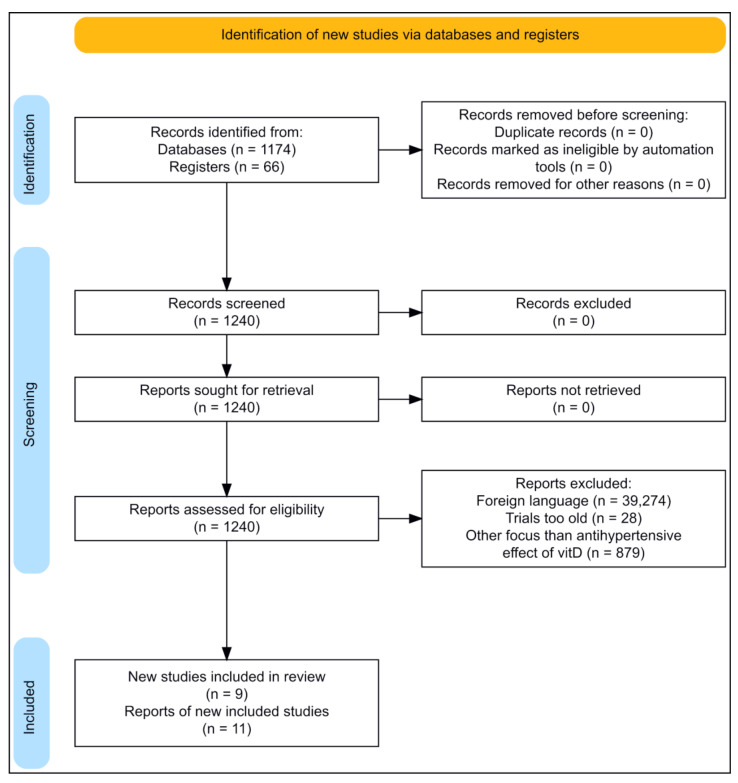
Flow diagram of the literature searched for this review. (Made with https://estech.shinyapps.io/prisma_flowdiagram/, accessed on 11 December 2022).

The literature searched for this review was found through online databases, clinical trials, and very few online webpages. The online databases include PubMed (https://pubmed.ncbi.nlm.nih.gov/, accessed on 11 December 2022), Scopus (https://www.scopus.com/search/form.uri?display=basic#basic, accessed on 11 December 2022), and Clinical Trials (https://clinicaltrials.gov/, accessed on 11 December 2022). Original papers were found from a systematic literature search using several different databases, and from written reviews. The searches were (if possible) restricted to be from 2017–2022, ensuring the most recent trials would be found; however, earlier trials have also been included. The search terms were ((“vitamin D”) OR (cholecalciferol)) AND (hypertension). Clinical trials in this review are included to assess the efficacy of vitD supplementation on hypertension. The search process is outlined in the PRISMA flow diagram (Figure 2).

## 3. Results and Discussion

### 3.1. Arterial Hypertension

Arterial hypertension (AH) is a common condition involving the arterial blood pressure (BP) being too high. This means that the force of blood pushing against the walls of the arteries is constantly elevated. This change affects the heart, which needs to work hard to pump sufficient amounts of blood in the body [8]. HT is traditionally defined as a persistent BP measured as ≥140/90 mmHg [9]. Maintenance of a normal BP is dependent on the balance between the cardiac output and the vascular resistance throughout the organism. Furthermore, the cardiac output is dependent on the stroke volume and heart rate (HR) [10].

Even though it is fairly impossible to find a clear underlying cause for most HT cases, there are still various risk factors that can lead to HT. Age, family history, obesity, lack of exercise, smoking, excessive salt diet, high alcohol consumption, and even pregnancy, are a few among the known risk factors responsible for the development of HT [8]. Besides lifestyle risk factors, prescribed drugs, such as oral contraceptives, non-steroidal anti-inflammatory drugs, ciclosporin, erythropoietin (EPO), and glucocorticoids (steroid hormones), may also raise the BP and induce HT [11].

Physiological maintenance of a normal BP is dependent on the balance between cardiac output and the vascular resistance of the system. There is an interchange between electrical, biochemical, and mechanical forces to control the BP. The electrical component is the sympathetic nervous system; the biochemical component is the renin–angiotensin–aldosterone system (RAAS), neurotransmitters (e.g., norepinephrine (noradrenaline)), or cytokines; and the mechanical component is the HR and the vasodilation/vasoconstriction of the arterioles. Thus, HT occurs when vascular regulation results from malfunctioning in the arterial BP control mechanisms of the body [10].

### 3.2. Forms of Hypertension

BP levels measured in the clinic may differ significantly when measured in an out-of-clinic setting by ambulatory BP monitoring (ABPM) or home BP monitoring [12]. White coat HT describes elevated clinical BP present in untreated individuals, but their out-of-office values are normal [13]. Masked HT is the inverse occurrence, characterized by an elevated out-of-clinic BP despite a normal clinic BP. The term masked HT can also be used as a term for people being treated for HT [12]. Resistant HT appoints to part of the population whose BP cannot be controlled. This means that despite treatment with the combination of three or more antihypertensive drugs at an adequate or full dosage, their BP values still remain above the therapeutic goals [14]. Table 1 gives an overview on the office BP classification and the definitions of the HT grade.

### 3.3. Health Risks and Mortality

HT is the third leading cause of the global burden of disease with 64 million disability-adjusted life years [16]. As populations live longer, adopt more inactive lifestyles, and become less economically burdened, it is estimated that the number of people with HT will increase by 15–20% by the year of 2025, reaching 1.56 billion hypertensive cases [17]. The overall prevalence of HT in adults globally is around 30–45% [18], which is an estimated 1.28 billion adults, with two-thirds living in low- and middle-income countries [19]. Pathologically increased BP is globally the strongest risk factor for cardiovascular disease (CVD) and related disability, despite extensive knowledge on the strategies to prevent and treat HT [20]. This becomes clearer with the fact that over the past 30 years, the disability-adjusted life caused by HT have increased by 40% since 1990 [21]. The largest number of systolic blood pressure (SBP)-related deaths are caused by ischemic heart disease (4.9 million), hemorrhagic stroke (2.0 million), and ischemic stroke (1.5 million) [21].

### 3.4. Normal Treatment of Hypertension

There are two well-established strategies to lower BP: lifestyle interventions and antihypertensive drug treatment [22]. The modifiable factors of HT include diets with excess salt consumption, high intake of saturated and trans fats, low intake of fruits and vegetables, physical inactivity, smoking, alcohol consumption, and being overweight or obese [19].

The Dietary Approaches to Stop Hypertension (DASH) trial proposes a diet that emphasizes fruit, vegetables, and low-fat diet products and is recommended in national guidelines [23]. It proved to lower BP substantially both in people without HT and with HT [23]. Exercise reduces the rate of progression from pre-hypertension to HT. Fitness in general reduces the risk of developing HT regardless of age, body mass index (BMI), and other traditional risk factors [24].

It is recommended to prescribe antihypertensive drugs in all patients with an SBP ≥ 140 mmHg or diastolic blood pressure (DBP) ≥ 90 mmHg, when the lifestyle adjustments are unsuccessful [25]. Referencing recent guidelines, five major drugs classes are recommended for the treatment of hypertension: angiotensin converting enzyme (ACE) inhibitors, angiotensin receptor blockers (ARBs), beta-adrenergic receptor blockers (BARBs), calcium channel blockers (CCBs), and diuretics [22]. Their mechanisms of actions are described in Table 2.

### 3.5. Vitamin D Metabolism

In human skin, vitD is synthesized by photoconversion of 7-dehydrocholesterol (7-DHC) to pre-vitamin D3 (Pre-D3). Pre-D3 then isomerizes to vitamin D3 (VD3) [36]. First, the B ring is broken by UV-light (280–320 nm) radiation from the sun, forming the Pre-D3. The isomerization to VD3 happens in a noncatalytic, thermo-sensitive process [37]. Once VD3 is formed, it is translocated into the circulation by the vitamin D-binding protein (VDBP). Thus, the VDBP ensures the efficient conversion of the quantitatively smaller amounts of Pre-D3 to VD3 by shifting the Pre-D3 ⇋ VD3 reaction to the right [37].

VitD is available in two distinct forms, cholecalciferol (D3) from animal sources and ergocalciferol (D2) from plant sources [38]. Studies show that cholecalciferol is much more effective than ergocalciferol in humans, because it appears that cholecalciferol raises and maintains 25-hydroxycholecalciferol levels to a substantially greater degree than ergocalciferol [39]. VitD is transported either within chylomicrons or bound to VDBP to the liver or is stored into body fat [40]. The hepatic entry of vitD is modulated by its plasma carriers, and when vitD enters the liver, it is hydroxylated into 25-hydroxycholecalciferol by the enzyme 25-hydroxylase [40,41]; 25-hydroxycholecalciferol is considered the most reliable marker of an individual’s vitD status [41]. After the hydroxylation, 25-hydroxycholecalciferol is transported from the liver to target tissues, primarily the kidney, where it is converted into 1,25-dihydroxyvitamin D3 (1,25(OH)_2_D_3_) by the enzyme 25-hydroxyvitamin D3-1α-hydroxylase, a mitochondrial P450 enzyme [40,42]. Cholecalciferol must be metabolized prior to initiation of its characteristic physiological response to the biologically active form 1,25(OH)_2_D_3_ [43]. 1,25(OH)_2_D_3_ plays an essential role in calcium and phosphate homeostasis, bone growth, and cellular differentiation [42].

### 3.6. Genomic and Non-Genomic Response of the Vitamin D Receptor

**Figure 3:**
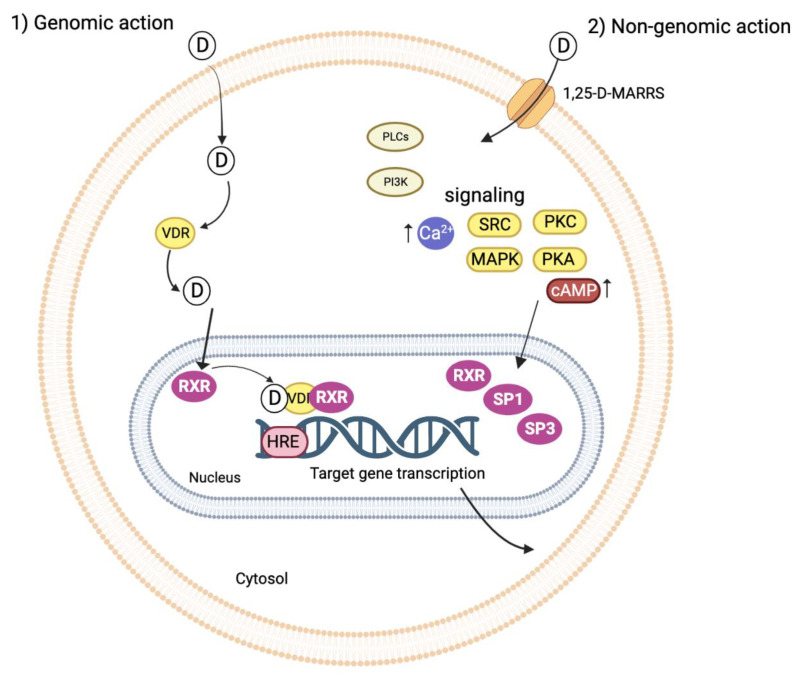
1,25(OH)_2_D_3_ genomic (1) and non-genomic (2) pathways. The VDR acts with other nuclear hormone receptors, especially the RXR [47]. 1,25(OH)_2_D_3_ crosses the cell membrane and enters the nucleus, where it interacts with the VDR. The VDR/1,25(OH)_2_D complex translocates into the nucleus in a ligand-dependent fashion [48]. In the nucleus, the VDR/1,25(OH)_2_D generates an active signal transduction complex consisting of the heterodimer of the vitamin D-liganded VDR and the RXR. The VDR/RXR heterodimer recognizes the HRE in the DNA sequence of the vitamin D-regulated genes [46]. Here, regulation of gene expression in specific tissues mediated by the VDR occurs, evoking the (1) genomic response. (2) The non-genomic actions are mediated by binding of calcitriol to the membrane located 1,25-D-MARRS, which affects numerous intracellular cell signaling pathways modulating the effects of gene expression through signal transduction to target transcription factors SP1, SP3, and RXR [51,52]. This figure was made using Biorender.com, agreement number XE24V0N4B7.

The VDR was first discovered as binding protein in the intestine [44]. Later, it was revealed to be active in the parathyroid gland, bone, pancreas, and kidney [45]. The hormone 1,25(OH)_2_D_3_ evokes both genomic and nongenomic responses [46]. In the genomic response, the VDR acts jointly with other nuclear hormone receptors, in particular the retinoid X receptor (RXR) [47]. During the hormone-induced receptor activation, the VDR translocates into the cell nucleus in a ligand-dependent fashion [48] and generates an active signal transduction complex consisting of a heterodimer of the 1,25(OH)_2_D_3_-liganded VDR and the RXR. This VDR–RXR heterodimer recognizes the vitD response elements in the DNA sequence of the vitD-regulated genes [46]. Transactivation by the liganded VDR/RXR is furthermore reliant upon the binding of one or more co-activator complexes, which permit the bridging to the RNA polymerase II machinery [49]. The mechanistic range of 1,25(OH)_2_D involves gene expression regulation in specific tissues, which is mediated by the nuclear VDR. The VDR is a DNA-binding protein that interacts directly with regulatory sequences near the target genes. It functions by recruiting chromatin-active complexes that participate in modification of both genetic and epigenetic transcriptional outputs [44,45,50]. An overview of the genomic and non-genomic pathways is given in Figure 3.

The non-genomic response to 1,25(OH)_2_D_3_ starts with the binding of calcitriol to its membrane VDR, and the 1,25D-membrane-associated rapid response steroid-binding protein (1,25-D-MARRS). This interaction between 1,25(OH)2D_3_ and 1,25-D-MARRS affects numerous cell signaling pathways via direct protein–protein interactions [51]. Examples of signaling pathways are the MAP kinases ERK1/ERK2/ERK5 and JNK MAP kinase modules [49,53]. Other studies also show that the rapid action of 1,25(OH)2D_3_ in intestinal epithelial cells is mediated by the activation of protein kinase C (PKC) [54]. Several other signaling molecules are also activated by the non-genomic response, such as phospholipase A_2_, phosphatidylinositol-3-kinase, and p21ras, as well as the rapid generation of second messengers, such as calcium, cyclic AMP, fatty acids, and 3-phosphoinositides [52]. Further downstream targets include transcription factors SP1, SP3, and RXR that bind to response elements on the promoters of vitD-responsive genes [52].

### 3.7. Functions of Vitamin D

1,25(OH)_2_D_3_ circulates to various target tissues to exert its actions, which are largely mediated by the nuclear VDR/RXR genomic actions [55]. The 1,25(OH)2D_3_ and VDR interaction regulates the expression of at least eleven genes encoding bone and mineral homeostasis effectors [56]. 1,25(OH)_2_D_3_ actively regulates at the transcriptional level the expression of calbindin, the calcium ATPase PMCA1b, and the renal TRPV5 and intestinal TRPV5 and TRPV6 calcium channels. These gene products all promote calcium absorption in the intestine and reabsorption in the kidney and thus enhance skeleton mineralization [56,57]. The homeostasis of serum inorganic phosphorus (P_i_) is primarily regulated by parathyroid hormone (PTH) and 1,25(OH)_2_D_3_ [58]; however, 1,25(OH)_2_D_3_ inhibits PTH release [59]. The intestinal phosphate absorption requires the Na^+^/phosphate cotransporter NaPi-2b/Slc34a2, and the expression of this channel is stimulated by 1,25(OH)_2_D_3_ [60]. Fibroblast growth factor (FGF23) also plays a role in phosphate homeostasis, a phosphaturic peptide which is expressed in the bone via stimulation of 1,25(OH)2D_3_ and inhibits renal Npt2a and Npt2c similar to PTH, eliciting phosphaturia [61].

**Figure 4:**
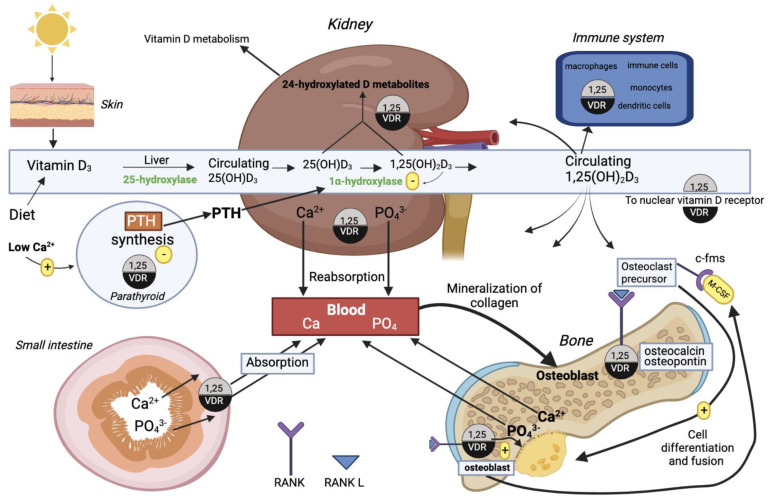
Effects of vitamin D and its metabolites in calcium homeostasis, bone metabolism, and the immune system. Vitamin D_3_ obtained from the diet or synthesized by photoconversion from sunlight is converted into the hormonal form 1,25(OH)_2_D_3_ by two hydroxylations occurring in the liver and kidney, respectively. After this conversion, 1,25(OH)_2_D_3_ circulates in blood to target tissues. The integrated actions of the 1,25(OH)_2_D_3_ hormone via binding to the intracellular VDR, to control calcium homeostasis in bone, kidney, intestine, and parathyroid, is shown above [64]. This figure was made using Biorender.com, agreement number UN24V0NHC0.

In terms of bone homeostasis, 1,25(OH)_2_D_3_ possesses both bone-resorbing and bone-remodeling properties. The bone-resorbing effect of 1,25(OH)2D_3_ is the ability to support osteoclastogenesis by upregulating the RANKL expression on osteoblasts/stromal cells [62]. In order for osteoclasts to differentiate into bone-resorbing osteoclasts, the RANKL/RANK signal also requires ligand binding of c-fms, an osteoclast precursor surface receptor present on the macrophage colony stimulating factor [55]. The bone-remodeling properties of 1,25(OH)_2_D_3_ is the synthesis of the bone matrix proteins osteopontin, collagen, and osteocalcin through transcriptional control in osteoblasts [56,63]. Figure 4 gives an overview of the functions of vitD in calcium homeostasis, bone metabolism, and the immune system.

1,25(OH)_2_D_3_ plays several roles outside of mineral and bone homeostasis. Moreover, the hormone is involved in modulating the immune response. Both the VDR and vitD-metabolizing enzymes are present in immune cells, monocytes, macrophages, dendritic cells, and activated B and T cells, which indicates that vitD exerts immunoregulatory effects on the innate and adaptive immune response [65].

Finally, the physiological effects of VDR activation in experimental studies include the suppression of neurohormonal activity and improvements in endothelial and vascular function [66]. In 1986 a study described a continuous relationship between 1,25(OH)_2_D_3_ levels and plasma renin activity (PRA) [67]. Another publication [68] reported a pronounced increase in ang II and renin in VDR-null mice. This suggests an interplay between the RAAS and VDR activation, which suggests that 1,25(OH)_2_D_3_ is directly involved in AH.

### 3.8. Vitamin D and Hypertension

As already mentioned earlier, HT has many etiological factors, vitD deficiency being one of them. The vitD level is inversely related to BP and incident HT [69]. Animal and human studies have suggested that the development of HT in individuals with lower levels of 1,25(OH)_2_D_3_ is due to the fact that the 1,25(OH)_2_D_3_ deficiency may increase activity of the RAAS, both systemically and in the kidney [70]. An increased plasma renin concentration (PRC) in a low 1,25(OH)_2_D_3_ setting may elevate sympathetic activity and enhance intra-glomerular pressure, predisposing to AH, a decline in GFR, and subsequent cardiovascular damage [71]. Knocking out of either the VDR or the 1α-hydroxylase gene in mice upregulates RAAS activity and induces HT [72,73], while treatment of these animals with 1,25(OH)_2_D_3_ suppresses the RAAS activity [73]. Additionally, the VDR is expressed in vascular tissues, including the myocardium, renin-producing juxtaglomerular cells, and vascular smooth muscle, where it directly influences calcium influx, muscle relaxation, and diastolic function [74,75].

Aside from the RAAS, vitamin D can exert its influence on hypertension through several other ways. As stated in 3.7, vitamin D is involved in calcium homeostasis by stimulating the production of calcium transporters, increasing calcium reabsorption in the kidney, and inducing the osteoclastic calcium release in bones [76,77]. Therefore, a vitamin D deficiency can result in a decreased concentration of Ca^2+^ in the plasma, which will lead to the secretion of parathyroid hormone (PTH) from the chief cells in the parathyroid gland to counteract this. Several epidemiological studies have shown that elevated PTH levels were associated with higher SBP and DBP values and a higher prevalence of HT in general [78,79,80]. These results were validated by the observation that PTH administration could induce elevated BP values in healthy subjects [81,82]. So far, the underlying mechanism has no yet been fully understood, and it is hypothesized that PTH-induced hypercalcemia might impair endothelial function [83].

Furthermore, vitamin D might have a direct effect on vascular stiffness. Both endothelial and vascular smooth muscle cells (VSMCs) express 1α-hydrolase, which is involved in the conversion of 25(OH)D to calcitriol [84]. It has been shown that this enzyme is activated in HUVECs by inflammatory molecules such as TNF-α and lipopolysaccharide [85]. In addition, exogenously added 1,25(OH)_2_D_3_ and 25(OH)D_3_ attracted monocytes and increased their binding to HUVECs [85]. In addition, it was found that vitamin D has a direct effect on vascular tone by reducing calcium influx [86]. Lastly, Richart et al. [87] proposed extrarenal activation of vitamin D as a possible contributor to hypertension and arterial stiffness. Macrophage vitamin D activation is much less tightly controlled than renal activation. In atherosclerotic lesions, they penetrate the arterial wall, and the activated vitamin can directly act on VSMCs. There, it can enhance the response to vasopressors [88], promote calcification [89], and induce cell dedifferentiation and oxidative stress [90].

Several observational studies have suggested a direct correlation of low plasma 25(OH)D concentrations and the risk of developing hypertension and hypertension-related complications [91,92,93,94,95,96,97,98,99,100,101,102,103]. Therefore, a supplementation with vitamin D seems a promising therapeutic option for these patients.

### 3.9. Clinical Trials with Vitamin D and Hypertension

Table 3 provides an outline of recent completed clinical trials treating participants with vitD either as a supplement to another antihypertensive agent or as an antihypertensive agent on its own. In addition, three trials studying vitamin D and immune mechanisms of hypertension in type II diabetics (VDIM, NCT03348280), fibroblast growth factor 23 and hypertensive disorders complicating pregnancy (NCT03821922), and vitamin D in pregnancy (GRAVITD. NCT04291313) are currently recruiting but have no results yet. Several other studies without NCT numbers have also been included in the discussion to broaden the amount of scientific evidence to give the best possible assessment of vitD as a potential antihypertensive supplement.

Overall, the effects of vitamin D supplementation on hypertension in the studies listed in Table 3 were negligible. They found no tangible evidence for an antihypertensive action of vitamin D alone. The recommended daily uptake of vitamin D lies between 200 and 600 UI/d [110], with 600 UI/d being advocated by most newer guidelines in order to achieve 25(OH)D serum concentrations of about 50 nmol/L [111]. No definitive recommendation exists for the upper limit of vitamin D supplementation; however, both the Institute of Medicine of the National Academies (IOM) as well as the European Food Safety Authority (EFSA) advise against doses exceeding 4000 UI/d to avoid hypercalcemia [112,113]. The dosages of the listed studies lay well within this range, some even at the higher limit, so the lack of effect cannot be attributed to underdosage. These findings are in accordance with a recent post-hoc analysis of the data from the Styrian Vitamin D Hypertension Trial of 2011–2014, where the authors also found no evidence of an antihypertensive effect of vitamin D supplementation [114].

It is possible that the underlying mechanisms of the association of low vitamin D plasma levels and hypertension are far more complicated as to be directly mediated by a vitamin D supplementation. Recent genetic studies showed that certain polymorphisms of the vitamin D receptor gene (*VDR*) and its downstream pathway genes were associated with the risk for HT. Caccamo et al. demonstrated an association between the *Fok I* and *Bsm I* SNPs with gestational hypertension [115]. *Fok I* was also associated with a higher incidence of heart failure and hypertension in patients with cardiovascular disease [116] and with the risk of developing essential hypertension [117], while *Bsm I* was closely linked to a higher predisposition to hypertension in pregnant women [118]. Vitamin D deficiency and the AA + AG genotype of the *Taq-I* SNP were linked to stage 2 hypertension in postmenopausal women [119]. In a study including 3699 pregnant women, polymorphisms in *CYP24A*1, *GC*, and *LRP2* genes were associated with blood pressure and hypertensive disorders of pregnancy [120]. So far, these studies were merely observational, and further research is warranted to elucidate possible mechanistic implications.

### 3.10. Discussion

Despite the numerous scientific correlations between vitD, RAAS activity, PRC, and HT, the BP-lowering effects of vitD replacement have not been observed in most studies, and only found to be effective in a few studies [69]. A randomized, double-blind, placebo-controlled trial involving 84 vitD-deficient, overweight participants without HT, treated the participants with 50.000 IU/week ergocalciferol for 8 weeks. This treatment showed no effect of vitD supplementation on RAAS activity or BP when compared to placebo [70]. Pilz et al. tested the effect of vitD supplementation on 188 hypertensive and viD-deficient patients by treating them with 2800 IU of VD_3_ per day for 8 weeks, also without any significant effect on BP and several CVD risk factors [107]. It was proposed that longer treatment periods might be necessary to observe a potential long-term effect of vitD supplementation on BP or RAAS activity [70,107], which was studied by Arora et al. They tested individuals with low vitD status and SBP of 120–159 mmHg. Participants were randomized to either high dose (4000 IU/d) or low-dose (400 IU/d) oral VD_3_ for 6 months. Still, no association between the change in vitD and the primary 24-h BP end point was found [108]. Even among individuals with larger increases in 25-hydroxyvitamin D, no noticeable trend towards a lower 24-h BP was observed [108]. Beveridge et al. performed a vitD supplementation meta-analysis, in which BP was reported. They included 46 clinical trials (4541 participants) in the trial-level meta-analysis, whereas individual patient data were obtained for 27 trials (3092 participants). At the trial level, no effect of vitD supplementation was seen on BP or SBP and DBP alone, thus leading to the conclusion that vitD supplementation is ineffective as an intervention for lowering BP [121].

Earlier trials have succeeded in showing the antihypertensive effects of vitD [122,123]. Panahi et al. conducted an open-label clinical trial involving 173 patients with essential hypertension, administering 50.000 IU/week vitD, and 1000 IU/day in patients with serum vitD levels < 20 ng/mL and 20–30 ng/mL, respectively, for 8 weeks. Eight weeks of supplementation with conventional antihypertensive drug regimens evoked a 5.5 ± 16.2 mmHg, 1.4 ± 12.6 mmHg, and 3.7 ± 9.2 mmHg decrease in the overall SBP, DBP, and MAP, respectively [124]. A single-center, double-blind, placebo-controlled trial by Chen et al. administered a conventional antihypertensive drug (nifedipine, 30 mg/d) to all participants. In total, 126 participants with graded 1–2 essential hypertension were randomly assigned to receive either vitD (63 participants, 2000 IU/d) or placebo (63 participants) for 6 months. The mean reductions in 24-h, daytime, and night-time mean SBP and DBP during ABPM were all significantly greater in the vitD supplementation group than in the control group after 6 months [125]. Another double-blind randomized clinical trial by Sheikh et al. administered conventional antihypertensive agents alongside vitD or placebo and showed similar antihypertensive effects of vitD [126].

The effect of vitD on the RAAS in mice showed promising results [72,73]. However, some human studies provided different results. A study involving 18 participants with type 2 diabetes (T2DM) administered calcitriol, a VDR agonist, or placebo for three weeks. No effects of calcitriol in terms of reducing RAAS activity or any significant altering of hemodynamic parameters, such as BP or renal plasma flow, was found [109]. Similarly, Bernini et al. showed that neither calcitriol nor VD_3_ therapy altered renin activity, circulating ang II, or aldosterone in hypertensives with vitD deficiency [127]. In contrast to these findings [109,127], Vaidya et al. showed a renal-vascular tissue RAAS-lowering effect when administering high dose cholecalciferol in non-diabetic, obese individuals with HT and vitD deficiency [128]. Other studies have been successful in showing the effects of cholecalciferol in lowering renin [105,129], and improving endothelial function [130] and plasma aldosterone [66].

Neither Pilz et al. nor Arora et al. [107,108] found any antihypertensive effects of vitD; however, they both agreed that vitD supplementation could be beneficial for other cardiovascular end points. This was tested in a double-blind, placebo-controlled trial involving 44 hypertensive patients by Qasemi et al. It aimed to examine the effect of vitD supplementation on flow mediated dilatation (FMD), oxidized LDL (O-LDL), and intracellular adhesion molecule 1 (ICAM1) [131]. These are inflammatory factors associated with CVD. In the vitD group, O-LDL and ICAM1 significantly decreased, while FMD increased in both groups. However, FMD was significantly higher in the vitD group [131], thus showing beneficial results for other cardiovascular end points than BP.

Bislev et al. treated 81 postmenopausal women with both ARBs with adjuvant vitD supplementation and with vitD alone for 2 weeks. ARBs proved to be BP reducing, while vitD supplementation alone did not affect BP, cardiac conductivity, or renin and aldosterone measurements [106]. Even though vitD might not currently be able to replace standard pharmacological treatment, according to recent trials and meta-analyses [70,107,108,121], it has proven to be a promising adjunct therapeutic agent, showing better antihypertensive results when administered alongside an antihypertensive medication [124,125,126,132,133].

Other positive effects of vitD include the correction of typical tissue sensitization to ang II induced by ACE-inhibitors, which was reported by one study [128]. Another study found that VD_3_ supplementation in patients with stable chronic heart failure may have additional benefits over direct renin inhibitors because renin inhibitors block the PRA, but it is at the expense of an increase in PRC, while VD_3_ may reduce both [105].

Concerning safety and potential adverse effects (AEs) of vitD supplementation, missing effects of most of their outcome variables show that vitD supplementation is relatively safe regarding many cardiovascular risk factors. VitD was safe concerning parameters of mineral/calcium metabolism [107]. The only statistically significant AE of vitD was an increase in triglycerides. However, considering that several previous RCTs did not measure increased triglycerides with vitD supplementation, Pilz et al. hypothesized that this elevation is not necessarily reflecting a true effect [107]. The DAYLIGHT study did not report any serious AEs during the 6 months of vitD supplementation, and none of the reported AEs were considered likely to be related to vitD supplementation [108].

The challenge remaining for the causal effects of vitD supplementation is acknowledging HT as a multifactorial disease, and that some individuals with other comorbidities, such as smoking, obesity, sedentary lifestyles, and metabolic syndromes, may have a lower threshold for vitD deficiency-induced HT compared to those without these comorbidities [134]. Further studies with larger study populations and a more granular stratification should be performed to further evaluate the role of vitD in HT [69].

## 4. Conclusions

HT is a multifactorial global disease that continues to grow in terms of the disease burden and prevalence. Both animal and human studies strongly support the hypothesis that vitD levels are inversely proportional to BP and incident HT, suggesting a potential role for vitD as an antihypertensive agent. However, studies examining this suggestion have produced mixed results. Most of the studies examining a direct effect of vitD on HT alone fail to show any significant BP decreasing effects, while supplementing vitD with another standard antihypertensive agent to potentiate the BP reduction showed promising results. Challenges remain in terms of proving a direct antihypertensive effect of vitD on its own, and further studies with higher doses, larger populations, and longer treatment periods are required to further evaluate the role of vitD in arterial hypertension.

## Figures and Tables

**Table 1:** Classification of office BP and definitions of hypertension grade.

Category	Systolic (mmHg)		Diastolic (mmHg)
Optimal	<120	And	<80
Normal	120–129	And/or	80–84
High normal	130–139	And/or	85–89
Grade 1 hypertension	140–159	And/or	90–99
Grade 2 hypertension	160–179	And/or	100–109
Grade 3 hypertension	≥180	And/or	≥110
Isolated systolic hypertension	≥140	And	<90

Table adapted from [15].

**Table 2:** Mechanisms of action of the different antihypertensive drug classes.

Antihypertensive Agent	Mechanism of Action
Diuretics	Diuretics produce a negative salt and water balance via similar pathways and prevent sodium retention in the long-term [26]. Several diuretics exist with different potencies and renal sites of action [26]. Loop diuretics (furosemide, bumetanide) exert their mechanism on NKCC transporters (NKCC1 and NKCC2), inhibiting the intracellular Cl^-^ concentration, and Na^+^ and Cl^-^ reabsorption in the renal tubule [27]. Thiazides act in the cortical diluting segment of the distal renal tubule, inducing diuresis by excreting mainly of sodium and chloride [28], inhibiting the thiazide-sensitive sodium-chloride cotransporter, which reabsorbs 5–10% of the filtered sodium load in the kidney [29].
ACE-inhibitors	RAAS is a key element in BP regulation and homeostasis of the body’s fluid volume. Renin breaks down angiotensinogen into angiotensin. In pulmonary blood vessels, angiotensin I interacts with ACE, which converts angiotensin I (Ang I) into the octapeptide hormone angiotensin II (Ang II) [30]. ACE-inhibitors prevent the conversion of Ang I to Ang II, thus inhibiting aldosterone release, promoting vasodilation [30].
Angiotensin receptor blockers	Ang II acts on the angiotensin II type 1 receptor (AT1R), which triggers vasoconstriction [30]. The AT1R is the principal regulator of BP and fluid volume homeostasis, hereby playing a vital role in cardiovascular and renal pathophysiology. Over-stimulation of the AT1R is implicated in hypertension. AT1R is the primary target receptor for the antihypertensive drug ARBs, therefore over-stimulation can be greatly reduced by treating with ARBs, thus reducing BP [31].
Beta-adrenergic receptor blockers	Stimulation of the β-adrenergic receptor (β-AR) by sympathetic neuronal activation (SNS), circulating catecholamines, or adrenergic agonists is proven to increase the heart rate, contraction force, and rate of cardiac relaxation [32]. β-AR can be divided into three subtypes, $\beta_{1}$-AR, $\beta_{2}$-AR, and $\beta_{3}$-AR, with the expression of mainly $\beta_{1}$-AR (75–80%) and $\beta_{2}$-AR in human cardiac tissue [32]. BARBs bind selectively to β-ARs, implementing competitive antagonism to the effects of the β-adrenergic stimuli [33]. Proposed antihypertensive functions of BARBs are [34]: Cardiac output reduction; RAAS inhibition; Plasma volume reduction; Reduction in the peripheral vascular resistance; Vascular compliance improvement; and Baroreceptor resetting.
Calcium channel blockers	Inhibit the flow of extracellular calcium by blocking ion-specific channels extending over the cell wall. Several types of ion-specific calcium channels have been identified; the mechanism of CCB action in humans is located in the L-type channels. Inhibiting calcium influx causes vascular smooth muscles to relax, evoking vasodilation and subsequently lowering the BP [35].

**Table 3:** Overview of recent completed clinical trials treating participants with vitamin D.

Title (Year of Completion)	Design	Intervention/Treatment	Endpoints/Outcomes	Results	Conclusions
Short-term vascular effects and oxidative status of calcium and vitamin D supplementation of postmenopausal hypertensive black women **NCT04255992** [104] (2020)	Double-arm, double-blind, randomized, and parallel clinical trial 22 participants	Patients with stable antihypertensive therapy for at least 3 months Calcium arm: 1000 mg calcium tablet per day for 8 weeks Vitamin D/calcium arm: 1000 mg/800UI of vitamin D/calcium tablet per day for 8 weeks	Primary: Change in 24 h BP profile from baseline to week 8 Secondary: Change in serum malondialdehyde concentration from baseline to week 8	Calcium vs. vitamin D/calcium arm: no significant differences in diurnal SBP reductions (4.7 [−2.5–10] vs. 4.7 [0.6–8.10] mmHg, *p* = 0.630) and HsCRP reductions (1.68 [0.43–5.58] vs. 1.46 [0.36–4.63] mg/L, *p* = 0.540). However, the vitamin D/calcium combination reduced uric acid levels significantly better than calcium alone (by 16 [9.63–24] vs. 11 [8–18] mg/L, *p* = 0.020). All values are given as median [interquartile range].	Supplementation with both calcium alone and calcium and vitamin D in postmenopausal hypertensive women is associated with a significant reduction of diurnal BP and inflammatory biomarkers.
Study to investigate the effects of vitamin D administration on plasma renin activity in patients with stable chronic heart failure (VitD-CHF) **NCT01092130** [105] (2013)	Open-label, blinded-endpoint, randomized, prospective trial 101 participants	Patients treated with ACE-inhibitors or ARBs and BARBs Control arm: no additional medication for 6 weeks Intervention arm: 2000 IU vitamin D daily, for 6 weeks	Primary: plasma renin activity (PRA) after 6 weeks Secondary: evaluation of the effect of vitamin D on plasma values of additional markers of RAAS activity, on different markers of the vitamin D cascade, on plasma levels of NT-proBNP, on urinary levels of markers of glomerular and tubular damage, on extracellular matrix markers, and on NYHA-class. Safety endpoints were biochemical indices of kidney function and bone homeostasis	Significant increase in both 25(OH)D and 1,25(OH)_2_D levels in the intervention group compared to control (80 [75–87] vs. 44 [39–49] nmol/L, *p* < 0.001 and 194 (179–211] vs.132 [121–143] nmol/L, *p* < 0.001, respectively) after 6 weeks. Significant decrease in both PRA and PRC in the intervention group compared to control (5.2 [2.9–9.5] vs. 7.3 [4.5–11.8] ng/mL, *p* = 0.002 and 55 [32–93] vs. 72 [47–111] ng/mL, *p* = 0.02, respectively) after 6 weeks. All values given as geometric means [95%CI].	Supplementation with dietary vitamin D_3_ (2.000 IU/d) in CHF patients increased vitamin D levels and lowered both PRA and PRC effectively compared to control.
Physiologic interactions between the adrenal- and the parathyroid glands **NCT02572960** [106] (2018)	Double-blind, placebo-controlled trial 81 participants	Patients with secondary hyperparathyroidism due to vitamin D deficiency Arm 1: Vitamin D_3_ 70 µg/day for 12 weeks valsartan 80 mg/day for 2 weeks Arm 2: Vitamin D_3_ 70 µg/day for 12 weeks placebo valsartan daily for 2 weeks Arm 3: Placebo vitamin D_3_/day for 12 weeks valsartan 80 mg/day for 2 weeks Arm 4: Placebo vitamin D_3_/day for 12 weeks placebo Valsartan daily for 2 weeks	Primary: Aldosterone at baseline and after 12 weeks of vitamin D_3_ treatment Secondary: PTH at baseline and after 2 weeks of ARB treatment, arterial stiffness, 24 h BP, physiological parameters	Valsartan (ARB) reduced DBP by −5.0 [−8.8; −1.0) mmHg vs. −2.6 [−7.5; 3.7] mmHg in the placebo group (*p* < 0.05). SBP was not significantly different in both groups. Renin increased strongly compared to placebo (81.2 [44.3; 180.0]) vs. 1.35 [−23.2; 46.3] pg/mL, *p* < 0.001), while the aldosterone ratio decreased significantly from valsartan treatment (−126 [−253; −52] vs. −8 [−59; 15], *p* < 0.0001). An addition of vitamin D_3_ had no further effect Vitamin D_3_ supplementation reduced PTH by −3.1 [−9.4; 9.1]% vs. a 5.7 [−5.2; 23.8]% increase in the placebo group (*p* = 0.1). All values are given as median [interquartile range].	No effect of ARB treatment on PTH plasma concentration. No correlation between calcium homeostasis and RAAS. Vitamin D_3_ supplementation reduced PTH, but did not affect BP, cardiac conduction, or the RAAS.
Effects of vitamin D on blood pressure and cardiovascular risk factors **NCT02136771** [66,107] (2014)	Single-center, double-blind, placebo-controlled, parallel-group study	Hypertensive patients with 25-hydroxyvitamin D levels below 30 ng/mL Treatment arm: 2800 IU vitamin D_3_ per day as oily drops for 8 weeks Placebo arm: Oily drops only as placebo for 8 weeks	Primary: 24-h systolic ambulatory blood pressure after 8 weeks Secondary: 24-h diastolic ambulatory blood pressure, plasma renin concentration, plasma aldosterone concentration, NT-pro-BNP, 24-h urinary albumin excretion, triglycerides, HDL-cholesterol	No significant reduction of 24 h SBP was observed (−0.4 [−2.8 to 1.9] mmHg, *p* = 0.712). Triglycerides increased significantly in the vitamin D group with a mean treatment of 17 [1–33] mg/dL (*p* = 0.013). A significant increase in 25(OH)D (mean treatment effect 11.5 [9.4–13.7] ng/mL: *p* < 0.001) and a significant decrease in PTH (−5.7 [−9.3 to −2.1] pg/mL; *p* = 0.003). All values given as mean [95%CI].	No significant effects of vitamin D supplementation on BP and several cardiovascular risk factors were shown; however, a significant increase in triglycerides was observed.
DAYLIGHT: Vitamin D therapy in individuals at high risk of hypertension **NCT01240512** [108] (2017)	Double-blind, randomized, controlled trial 534 participants	Patients with 25-hydroxyvitamin D <25 ng/mL, SBP 120–159 mmHg, DBP ≤ 99 mmHg, no antihypertensive medication High dose arm: 4000 IU/d vitamin D_3_ for 6 months Low dose arm: 400 IU/d vitamin D_3_ for 6 months	Primary: change in 24 h SBP after 6 months Secondary: change in 24 h DBP, change in mean daytime and nighttime ambulatory systolic and diastolic blood pressure, change in mean clinic systolic and diastolic blood pressure, change in mean clinic pulse pressure, all after 6 months	There was no significant difference in the primary end point or in any of the secondary end points. No evidence of association between change in 25-hydroxyvitamin D and change in 24-h systolic blood pressure after 6 months.	Supplementation with vitamin D did not reduce BP in vitamin D deficient individuals with either prehypertension or stage 1 hypertension.
The VALIDATE-D study **NCT01635062** [109] (2017)	Randomized, double-blinded, and placebo-controlled study 18 participants	Patients with treated type-two diabetes, normal blood pressure or stage 1 hypertension (treated or untreated), and normal kidney function Intervention arm: calcitriol (titrated up to 0.75 µg/d) for 3 weeks Placebo arm: placebo for 3 weeks	Primary: change in circulating RAS activity after 2 weeks Secondary: change in renal plasma flow and urinary protein excretion after 2 weeks	Increases in 1,25(OH)_2_D (45.4 ± 18.2 to 61.8 ± 11.2 pg/mL, *p* = 0.03) with calcitriol administration vs. no change in the placebo group. No significant differences in PRA, serum or urinary aldosterone, baseline ang II-stimulated MAP, or basal and ang II-stimulated RPF between interventions was found. Values are given as mean ± SD.	Calcitriol raises 1,25(OH)_2_D levels compared to placebo, but this has no significant effect on the change in circulating RAS activity or vascular hemodynamics.

## Data Availability

Not applicable.

## Abbreviations

1,25(OH)_2_D_3_	1,25-dihydroxyvitamin D3
1,25-D-MARRS	1,25D-membrane-associated rapid response steroid-binding protein
7-DHC	7-dehydrocholesterol
AB	Antibody
ABPM	Ambulatory Blood pressure measurement
ACE	Angiotensin converting enzyme
AE	Adverse effects
AH	Arterial hypertension
Ang I	Angiotensin I
Ang II	Angiotensin II
ARB	Angiotensin receptor blocker
AT1R	Angiotensin II type 1 receptor
B-AR	Beta-adrenergic receptor
BARB	Beta-adrenergic receptor blockers
C39	C. elegans miR-39
CCB	Calcium channel blocker
CTGF	Connective Tissue Growth Factor
CVD	Cardiovascular disease
DBP	Diastolic blood pressure
EMT	Epithelial to Mesenchymal Transition
FGF23	Fibroblast growth factor
FMD	Flow mediated dilatation
HR	Heart rate
HT	Hypertension
ICAM1	Intracellular adhesion molecule 1
O-LDL	Oxidized LDL
P_i_	Serum inorganic phosphorus
PKC	Protein kinase C
PRA	Plasma renin activity
PRC	Plasma renin concentration
Pre-D3	Pre Vitamin D3
PTH	Parathyroid hormone
RAAS	Renin-angiotensin-aldosterone-system
RXR	Retinoid X receptor
SBP	Systolic blood pressure
SH	Secondary hypertension
SNP	Single nucleotide polymorphism
T2DM	Type 2 diabetes
VD3	Vitamin D3
VDBP	Vitamin D-binding protein
VDR	Vitamin D receptor
VitD	Vitamin D
